# The Diagnostic Accuracy of Linear Endoscopic Ultrasound for Evaluating Symptoms Suggestive of Common Bile Duct Stones

**DOI:** 10.1155/2016/6957235

**Published:** 2016-08-17

**Authors:** Min Wang, Xu He, Chuan Tian, Jian Li, Feng Min, Hong-yan Li

**Affiliations:** ^1^Development of Gastroenterology, The People's Hospital of Nanchuan, Chongqing 408400, China; ^2^Clinical Medical Experimental Teaching Center, School of Clinical Medicine, Chengdu Medical College, Chengdu, Sichuan 610500, China

## Abstract

*Background*. In order to assess the diagnostic accuracy of linear EUS for evaluating clinically suggestive CBD stones in high-risk groups.* Methods*. 202 patients with clinically suggestive CBD stones in high-risk groups who underwent linear EUS examination between January 2012 and January 2015 were retrospectively reviewed. Endoscopic retrograde cholangiopancreatography (ERCP) with stone extraction or surgical choledochoscopy was only performed when a CBD stone was detected by linear EUS. Cases that were negative for CBD stones were followed up for at least 6 months.* Results*. Of 202 enrolled patients, 126 were positive for CBD stones according to linear EUS findings. 124 patients successfully underwent ERCP, and ERCP failed in 2 who were later successfully treated by surgical intervention. There were 2 false-positive cases with positive findings for CBD stones on ERCP. Among 76 patients without CBD stones, no false-negative cases were identified during the mean 6-month follow-up. Linear EUS had sensitivity, specificity, and positive and negative predictive values for the detection of CBD stones of 100%, 92.88%, 98.21%, and 100%, respectively.* Conclusions*. Linear EUS is a safe and efficacious diagnostic tool for evaluating clinically suggestive CBD stones with high risk of choledocholithiasis. Performing linear EUS prior to ERCP in patients with symptoms suggestive of CBD stones can reduce unnecessary ERCP procedures.

## 1. Introduction

Common bile duct (CBD) stones are a common clinical problem; however, their etiology is not well understood, and they can cause serious complications [1]. It is usually difficult to confirm choledocholithiasis when the symptoms are only clinically suggestive of this condition. Endoscopic retrograde cholangiopancreatography (ERCP) with endoscopic sphincterotomy is the gold standard for diagnosing and treating CBD stones. Yet, the complication rate associated with ERCP ranges from 5 to 10%, and the rate of post-ERCP pancreatitis is about 15% [2, 3].

EUS is a minimally invasive procedure for evaluating CBD stones, and it has a diagnostic accuracy comparable to ERCP [4]. EUS can reduce unnecessary ERCP procedures and prevent post-ERCP pancreatitis and other ERCP-related complications [5]. Most of reports about EUS in the literature focus on radical EUS, but there are few reports on using linear EUS to evaluate symptoms clinically suggestive of CBD stones [6, 7]. We report our experience with the diagnostic accuracy of linear EUS for evaluating symptoms suggestive of CBD stones which fulfill the criteria for a high likelihood of choledocholithiasis [8].

## 2. Materials and Methods

This retrospective study was approved by the institutional review board of The People's Hospital of Nanchuan, and written informed consent was obtained from all the patients. Two hundred and two patients who were considered to have CBD stones between January 2012 and January 2015 were enrolled.

Inclusion criteria were as follows: patients with at least two manifestations of acute upper abdominal pain with or without gallstones prior to admission; an unexplained derangement of serum liver biochemical tests (such as abnormal levels of aspartate aminotransferase, alanine aminotransferase, total bilirubin, alkaline phosphatase, and *γ*-glutamyl transpeptidase); an enlarged CBD stone ≥6 mm with an intact gallbladder or a CBD stone ≥8 mm in patients who had undergone cholecystectomy under transabdominal ultrasound of the upper quadrant without an identifiable cause; conventional ultrasound or computed tomography (CT) findings suggestive of CBD stones; and those with biliary pancreatitis. Suspected CBD stone was classified as high risk according to standard guidelines, but without CBD stone detected in transabdominal ultrasonography or CT images. High-risk predictors according to the guidelines include CBD stone on transabdominal US, or clinical ascending cholangitis, bilirubin 0–4 mg/dL, dilated CBD on US (0–6 mm with gallbladder in situ), or bilirubin level 1.8–4 mg/dL [8].

Exclusion criteria were patients with a history of sphincterotomy or biliodigestive surgical anastomosis; those with a previous history of a pancreatobiliary disease such as chronic pancreatitis; patients with a history of CBD stones that were accurately diagnosed by ultrasound or CT; abnormal liver function test results due to drug or alcohol consumption; those with a tumor of the bile duct that had already been identified; and patients with a severe cardiovascular or psychiatric disease.

Linear EUS was performed with a linear EUS device (SU7000, EG-530UT, Fujifilm, Tokyo, Japan) at a frequency of 5.0 MHz. Linear EUS was performed with the patient in the left lateral decubitus position, and local anesthesia of the pharynx (xylocaine) was administered, and an intramuscular injection of raceanisodamine hydrochloride (10 mg) and meperidine (100 mg) was administered as premedications. The patients were monitored during linear EUS of the stomach, duodenal bulb, and descending duodenum. A CBD stone was diagnosed by linear EUS if a persistent hyperechoic lesion was found with an acoustic shadow.

In cases that were positively diagnosed with CBD stones by linear EUS, ERCP was performed within the next 3 days. ERCP was performed with a duodenoscope (ED-250XL5, Fujifilm). Cholangiography and endoscopic sphincterotomy were recommended for patients with CBD stones diagnosed by linear EUS, and the stones were removed by using a Dormia basket or balloon-tipped catheter. If ERCP failed, surgical intervention was subsequently performed. If the patients were negative for CBD stones according to linear EUS findings, they were followed up for at least 6 months to monitor their liver biochemical tests or CT, magnetic resonance cholangiopancreatography (MRCP), or EUS results. Experienced interventional endoscopists performed linear EUS and ERCP.

Results of the extracted CBD stones from ERCP or surgery were used as the diagnostic gold standard. The diagnostic accuracy of linear EUS compared to the gold standards was assessed in terms of the accuracy, sensitivity, specificity, positive predictive value, and negative predictive value.

## 3. Results

Between June 2012 and December 2015, 202 patients with symptoms clinically suggestive of CBD stones were enrolled in this study. Patients' characteristics are shown in Table 1. There were 118 men and 84 women (mean age: 59.6 ± 13.2 years). One hundred sixty-seven patients had an intact gallbladder, and among them, 29 had GB stones. The main reasons for inclusion were upper abdominal pain (168 patients), US or CT findings suggestive of CBD stones (68), a total bilirubin level ≥4 mg/dL (56), dilated CBD stones (59), and biliary pancreatitis (29).

**Figure 1:**
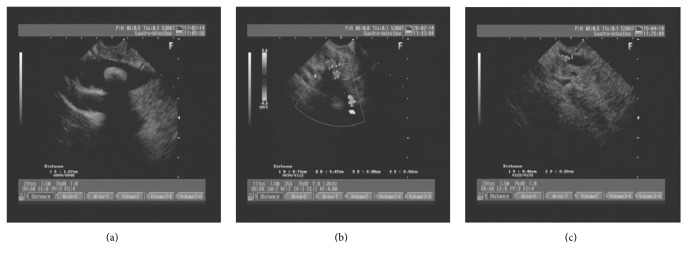
The diameter of common bile duct stones measured on linear endoscopic ultrasound. (a) >1 cm, (b) 0.5–1.0 cm, and (c) <0.5 cm.

**Figure 2:**
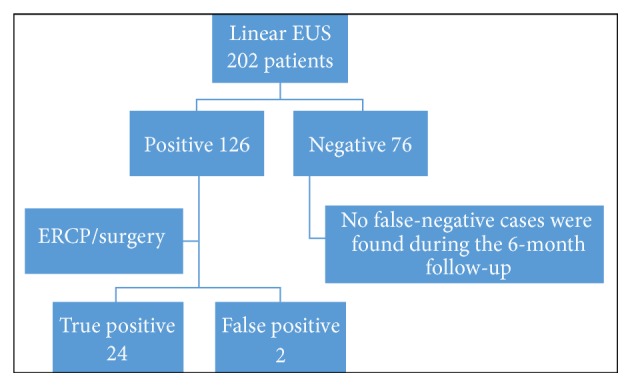
Patient flow chart. One hundred twenty-six patients were positive for common bile duct stones according to linear endoscopic ultrasound (EUS) findings. Of 76 patients, there were no false-negative cases identified during the 6-month follow-up. ERCP, endoscopic retrograde cholangiopancreatography.

Results of the linear EUS compared to those from ERCP or surgery are presented in Table 2. CBD stones were confirmed by linear EUS in 126 patients. The diameters of the CBD stones were >1 cm in 25 cases, between 0.5 cm and 1.0 cm in 79, and <0.5 cm in 22 cases (Figure 1). Of 126 patients who were positive for CBD stones, 124 were treated with ERCP, and of those, 122 had their CBD stones successfully extracted. ERCP failed in 2 patients who were later treated with surgery, and then the stones were successfully removed (Figure 2). There were 2 false-positive cases.

All 202 patients successfully underwent linear EUS by the same experienced endoscopist (W. M.), and no complications occurred after the procedure. After the ERCP procedure, 12 cases had complications, including 5 with minor ERCP-related pancreatitis, 3 with increased liver enzymes and a serum bilirubin level, 2 with septicemia, and 2 with postsphincterotomy bleeding (Table 2). All 12 patients had a perforation that was treated, and they made a full recovery. No patients died of severe ERCP-related complications.

Among 76 patients with negative linear EUS findings for CBD stones, no false-negative cases were found during the mean 6-month follow-up period (range: 1–12 months). The overall sensitivity, specificity, positive predictive value, and negative predictive value of the linear EUS for detecting CBD stones were 100%, 92.88%, 98.21%, and 100%, respectively.

## 4. Discussion

Spiral CT, MRCP, and EUS can be used instead of biliary imaging to diagnose CBD stones. The sensitivity, specificity, and accuracy of spiral CT for diagnosing CBD stones range from 85 to 88%, 88 to 97%, and 86 to 94%, respectively [9, 10]. Spiral CT is not superior to EUS and MRCP, as they are precise, minimally invasive diagnostic tools that are the first-choice method for diagnosing CBD stones [11, 12]. However, compared to EUS, the rate of detecting CBD stones with MRCP is associated with the size of the stone. For stones <5 mm, the sensitivity of MRCP is about 65%, and it is difficult to diagnose stones located in the region of Vater's papilla [11, 13]. A stone in the CBD appears as a hyperechoic focus with acoustic shadowing on EUS imaging, and there is clear boundary between the stones and the bile duct wall. EUS combined with an endoscope and high-resolution ultrasonic imaging is effective for avoiding the affection of the gas inside the lumen and the abdominal fat to obtain a clear image, which ultimately improves the accuracy of diagnosing CBD stones. Although there is no statistically significant difference between EUS and MRCP in terms of diagnosing CBD stones, the MRCP is more costly and it cannot be used in patients with any metal fragments in their body, such as an artificial cardiac pacemaker. In our study, EUS had a high sensitivity and specificity for detecting the retained CBD stones. Therefore, EUS is better than MRCP for detecting CBD stones. In addition, EUS is superior to MRCP for diagnosing small stones, especially those <0.5 cm, and acute biliary pancreatitis [11, 13, 14]. Linear EUS is similar to radical EUS on examination of common bile duct stone. But linear EUS can be guider for EUS-guided fine needle aspiration (FNA) for pathological and cytological diagnosis, for example, common bile duct stone and tumor. Meanwhile, linear EUS could be therapeutic applications for pancreatic abscess, pseudocyst drainage, gallbladder drainage, celiac plexus neurolysis block, and so on [15]. This can expand the usage of linear EUS in developing area in China. Otherwise, there are disadvantages for linear EUS. Linear EUS had higher requirements for operators, especially for freshman, for application on diseases of gall bladder and pancreas, for example, EUS-FNA. In our study, linear EUS diagnosed CBD stones in 126 cases, and the diagnosis was confirmed in 124 cases. ERCP confirmed CBD stones <5 mm in 22 patients. ERCP is the gold standard method for diagnosing CBD stones; however, this invasive test is associated with potential complications [16, 17]. The sensitivity and specificity of EUS for diagnosing CBD stones range from 89 to 94% and 94 to 95%, respectively [4, 18]. EUS can also reduce unnecessary invasive tests in cases that only have symptoms suggestive of CBD stones. The risk of complications with EUS-guided ERCP has significantly decreased, and this is probably because the ERCP procedure is not used in two-thirds of patients [5]. By performing linear EUS, 62.38% (126/202) of cases were found to be positive for CBD stone, and of those, 124 were clinically confirmed to have CBD stones. The remaining 76 patients without CBD stones who underwent clinical follow-up were all correctly diagnosed by linear EUS, which prevented unnecessary ERCP procedures. No complications occurred after the linear EUS procedures.

The reported sensitivity and specificity of radial EUS are the same as those of ERCP for detecting CBD stones. However, there are few reports on using linear EUS to assess CBD stones. In hospitals with limited funding and low quality MRI, linear EUS can be used to diagnose biliary tree disease, which can reduce the delay between the initial clinical presentation and the EUS examination. In our series, the sensitivity, specificity, positive predictive value, and negative predictive value of linear EUS for detecting CBD stones in the biliary tract were high. These results are in agreement with those of previous reports [4–7]. This suggests that linear EUS is diagnostically suitable for evaluating CBD stones in cases with clinically suggestive symptoms.

Our data confirmed that linear EUS is a noninvasive, safe, accurate imaging method for diagnosing clinically unconfirmed CBD stones in high risk of choledocholithiasis, and it can prevent unnecessary ERCP procedures.

## Figures and Tables

**(a):** 

Variable	Number
Sex, *n* (M/F)	118/84
Age, years (mean ± SD)	59.6 ± 13.2
Intact GB (GB stones)	167 (29)

**(b):** 

The main reasons for inclusion	Number of patients
Upper abdominal pain	168
US/CT findings suggestive of CBD stones	68
Biliary pancreatitis	29
Total bilirubin level ≥4 mg/dL	56
Dilated CBD	59

M, male; F, female; SD, standard deviation; GB, gallbladder; US, ultrasound; CT, computed tomography; CBD, common bile duct.

**Table 2:** The complications of linear EUS and ERCP.

	Complication	Number of complications	Percentage
EUS	None	0	0% (0/202)
ERCP		12	9.68% (12/124)
	Pancreatitis	5	4.03% (5/124)
	Increased liver enzymes	2	1.61% (2/124)
	Increased serum bilirubin	1	0.81% (1/124)
	Septicemia	2	1.61% (2/124)
	Postsphincterotomy bleeding	2	1.61% (2/124)

EUS, endoscopic ultrasound; ERCP, endoscopic retrograde cholangiopancreatography.
